# WiFi-based non-contact human presence detection technology

**DOI:** 10.1038/s41598-024-54077-x

**Published:** 2024-02-13

**Authors:** Yang Zhang, Xuechun Wang, Jinghao Wen, Xianxun Zhu

**Affiliations:** 1 School of Economics and Management, Shanghai Polytechnic University, Shanghai, 201209 China; 2 School of Electrical and Electronic Engineering, Hubei University of Technology, Wuhan, 430068 China; 3 School of Computer Science, Central China Normal University, Wuhan, 430079 China; 4 School of Communication and Information Engineering, Shanghai University, Shanghai, 200444 China

**Keywords:** Non-contact, Human presence sensing, Wireless perception, Machine learning, Signs and symptoms, Energy science and technology

## Abstract

In the swiftly evolving landscape of Internet of Things (IoT) technology, the demand for adaptive non-contact sensing has seen a considerable surge. Traditional human perception technologies, such as vision-based approaches, often grapple with problems including lack of sensor versatility and sub-optimal accuracy. To address these issues, this paper introduces a novel, non-contact method for human presence perception, relying on WiFi. This innovative approach involves a sequential process, beginning with the pre-processing of collected Channel State Information (CSI), followed by feature extraction, and finally, classification. By establishing signal models that correspond to varying states, this method enables the accurate perception and recognition of human presence. Remarkably, this technique exhibits a high level of precision, with sensing accuracy reaching up to 99$\%$. The potential applications of this approach are extensive, proving to be particularly beneficial in contexts such as smart homes and healthcare, amongst various other everyday scenarios. This underscores the significant role this novel method could play in enhancing the sophistication and effectiveness of human presence detection and recognition systems in the IoT era.

## Introduction

In contemporary society, the observation and interpretation of human activities carry substantial societal value. The choice of appropriate technologies for human perception has emerged as a significant area of modern research^1–3^. Conventional human perception technologies mainly include vision^4^, sensors^5^, and infrared^6^. Vision-based methods entail collecting images or videos through a camera, followed by the application of image processing algorithms for recognition and perception. Despite its high accuracy, this technique is notably vulnerable to lighting conditions’ variability, and the capture of video images may encroach on individual privacy. The sensor-based approach requires users to constantly wear sensors, potentially causing interruptions in their daily activities. Infrared-based methods, employing infrared sensors for human perception, are plagued by a high frequency of false alarms and are easily obstructed.

In 2011, aiming to surmount the limitations of traditional technologies, Halperin et al.^7^ developed a WiFi device firmware based on the IEEE 802.11n standard. This pioneering effort aimed to simplify the collection of CSI, laying the groundwork for human perception recognition. Subsequent to this advancement, various research groups and scholars have thoroughly explored CSI-based human perception technologies^8,9,24,25^. In 2016, the WiFi ID method proposed by Zhang et al.^10^ utilized Fourier transformation and the relief algorithm to extract gait information, performing gait recognition via the Support Vector Machine (SVM) algorithm and achieving an average accuracy rate of $93\%$. In 2017, Shi et al.^11^ executed human identification using a neural network model, attaining an accuracy rate of $94\%$. Despite the swift progression in the theoretical foundation and practical application of wireless sensing technology^12,13^, significant challenges such as inadequate robustness, low accuracy, and limited universality, continue to prevail^26–28^. This paper introduces a cutting-edge, non-contact human presence detection technology based on wireless sensing. The interpretation of the gathered sensing information allows for the identification of individuals within the sensing area, thereby enabling high-precision, non-contact sensing.

## System block diagram

**Figure 1:**
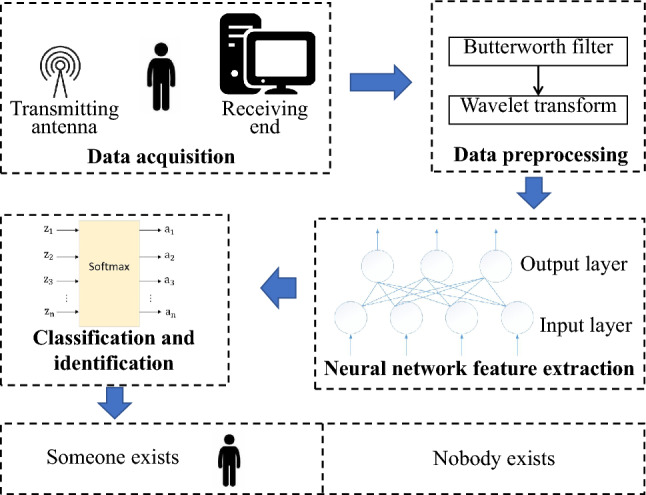
System block diagram.

The block diagram of the WiFi-based non-contact human presence sensing system proposed in this paper is presented in Fig. 1. Initially, a network card with modified firmware is utilized to gather channel state information. Following this, noise is mitigated using a low-pass filter and wavelet transform. Subsequently, an algorithm founded on a neural network is employed to extract distinguishing features. Ultimately, machine learning techniques are used to classify and identify human states.

## Relevant theories

According to the IEEE 802.11 standard, the process of WiFi signal transmission is segmented into various subcarriers, each operating at a distinct frequency. The propagation paths of these subcarriers through the environment differ, leading to the diversity observed in the CSI data^14^. The CSI encompasses a range of data, including the time delay, amplitude attenuation, and phase shift experienced during the signal’s transmission and reception. Essentially, CSI represents the physical layer information of the subcarriers, characterizing the cumulative effect on wireless signals at the receiver after undergoing reflection, refraction, and scattering across different environmental paths^29^.

In the frequency domain, the communication model for a system employing multiple transmitting and receiving antennas, characterized by narrowband flat fading, is represented as follows:

1 $$\begin{aligned} y = Hx + n \end{aligned}$$

Here, *y* denotes the received signal, *x* symbolizes the transmitted signal, *n* represents noise, and *H* is the signal transformation matrix. This matrix *H* reflects the impact of the external physical environment on the transmitted signal *x*, as it propagates from the transmitter to the receiver, transforming into *y*. The matrix *H* can also be estimated using the following equation:

2 $$\begin{aligned} {\hat{H}} = \frac{y}{x} \end{aligned}$$

CSI is essentially a representation of *H*. At the receiver, the CSI for each subcarrier can be quantified in terms of amplitude and phase, as per the equation:

3 $$\begin{aligned} H_i = \Vert H_i\Vert \exp (j\angle H_i) \end{aligned}$$

In this notation, $H_i$ signifies the value of the $i\textrm{th}$ subcarrier in the Channel State Information, $\Vert H_i\Vert$ represents the amplitude of the $i\textrm{th}$ subcarrier, and $\angle H_i$ denotes the phase of the $i\textrm{th}$ subcarrier^30^. The Signal-to-Noise Ratio (SNR) plays a critical role in this context, as it quantifies the level of the desired signal relative to the background noise, which is crucial in analyzing the quality and reliability of the communication channel. Variations in the environmental conditions during the wireless signal transmission can lead to multipath propagation, encompassing a line-of-sight path and several paths involving reflection and refraction^15,22^. The crux of this paper is to analyze the alterations in channel propagation, induced by environmental changes during the propagation process, to facilitate the detection of human presence.

## Methodology

### Pretreatment

The channel state information acquired directly is susceptible to low-pass noise and, therefore, cannot be directly employed for human presence detection. Accordingly, a low-pass filtering method is chosen^20^. This technique exploits the characteristics of inductors with high resistance at low frequencies and capacitors with low resistance at high frequencies to perform data denoising. The formula is as follows:

4 $$\begin{aligned} f_c=\frac{1}{2\pi RC} \end{aligned}$$

5 $$\begin{aligned} L_(s)=\frac{2\pi f_c}{2\pi f_c +s} \end{aligned}$$

where *R* is the resistance, *l* is the load series inductor, *C* is the parallel capacitor at both ends of the load resistance, and $f_c$ is the cutoff frequency.

To augment the detection of indoor human activities, wavelet transforms^17^, recognized for their aptitude to differentiate high and low-frequency components, are employed for noise reduction. The core principle hinges on signal extraction through localized transformations in both spatial and frequency domains. By employing scaling and translation, the CSI is analyzed multi-scale. This approach distinctly segregates high and low-frequency components, enhancing the robustness and precision of the sensing technology.The mathematical representations of the hard$(\phi _{\lambda \text {hard}}(\varpi ))$ and soft ($\phi _{\lambda \text {soft}}(\varpi )$) thresholding functions are shown in Eqs. (6) and (7), respectively.

6 $$\begin{aligned} \phi _{\lambda \text {hard}}(\varpi )=\left\{ \begin{array}{ll} \varpi , &{} {\mid \varpi \mid } {\ge } \lambda \\ 0, &{} {\mid \varpi \mid } < \lambda \\ \end{array} \right. \end{aligned}$$

7 $$\begin{aligned} \phi _{\lambda \text {soft}}(\varpi )=\left\{ \begin{array}{ll} \text {sgn}(\varpi )({\mid \varpi \mid }-\lambda ), &{} {\mid \varpi \mid } {\ge } \lambda \\ 0, &{} {\mid \varpi \mid } < \lambda \\ \end{array} \right. \end{aligned}$$

where $\varpi$ is the wavelet coefficient, $\lambda$ the threshold value, and $\text {sgn}(.)$ denotes the signum function. In hard thresholding, the coefficient is nullified if its absolute value is below the threshold, while preserved otherwise. In contrast, the soft thresholding reduces the absolute value of each coefficient by $\lambda$ and sets it to zero if the result is non-positive.

### Feature extraction

While preprocessing significantly improves signal quality, the extensive volume of data complicates the direct classification and interpretation of human activities. A self-organizing neural network^18^, utilizing unsupervised learning with a competitive approach, skillfully extracts channel state information features following preprocessing. This network primarily consists of input and output layers, dedicated to classification and clustering tasks. The operation sequence is as follows:

The data first undergoes normalization to facilitate uniformity in subsequent processes. This step is mathematically represented as:

8 $$\begin{aligned} {\hat{X}}=\frac{X}{\Vert {X} \Vert } \end{aligned}$$

Post-normalization, a similarity metric identifies the most relevant neurons. This involves the normalization of the weight vectors, expressed as:

9 $$\begin{aligned} \hat{W_j}=\frac{W_j}{\Vert {W_j}\Vert } \end{aligned}$$

where ${\hat{X}}$ and $\hat{W_j}$ denote the normalized input and weight vectors, respectively.

The core of the competitive learning algorithm is to minimize the distance between the input vector and the weight vector of the selected neuron. This is encapsulated by the following equation:

10 $$\begin{aligned} \Vert {{\hat{X}}} -\hat{W_{j^*}}\Vert =\min _{j\in (1,2,3,\ldots ,n)} \{ \Vert {{\hat{X}}} -\hat{W_{j}}\Vert \} \end{aligned}$$

where $j^*$ represents the index of the winning neuron.

Subsequently, the output values are updated as follows:

11 $$\begin{aligned} y_j(t+1)=\left\{ \begin{array}{ll} 1, &{} \text {if } j = j^*\\ 0, &{} \text {if } j \ne j^*\\ \end{array} \right. \end{aligned}$$

where $y_j(t+1)$ denotes the output of the $j\textrm{th}$ neuron at time $(t+1)$.

For the winning neuron, the weight vector is refined using the equation:

12 $$\begin{aligned} \hat{W_{j^*}}(t+1)=\hat{W_{j^*}}(t)+\alpha \left( {{\hat{X}}} -\hat{W_{j^*}}\right) \end{aligned}$$

Here, $\hat{W_{j^*}}(t)$ is the weight vector at time *t*, adjusted to reduce the disparity with the input vector ${{\hat{X}}}$. The term $\alpha$, representing the learning rate, falls within the range (0, 1]. It regulates the adaptation pace and magnitude of the weight vectors.

As the iteration count *t* progresses, the learning rate $\alpha$ gradually reduces to zero. This decline ensures the convergence and stability of the learning algorithm, thus preventing any potential overshooting of the optimal weight configuration. This methodical reduction in $\alpha$ is pivotal for the efficacy and efficiency of the learning process^16^.

### Classification

In the pursuit of classifying and discerning features indicative of human presence, this study employs a softmax classifier^19^. This classifier adeptly computes probabilities for various states corresponding to different feature vectors. The operational flow of the softmax classifier is outlined as follows:

Initially, data is introduced into the input layer. It then traverses through two distinct feature layers, undergoing processing and transformation. Conclusively, the softmax function is applied, ensuring that each output is normalized to a probability range of 0 to 1. This normalization is formally represented as:

13 $$\begin{aligned} \text {Softmax}(x)_i = \frac{e^{x_i}}{\sum _{j=1}^{K} e^{x_j}} \end{aligned}$$

for an input vector $x \in {\mathbb {R}}^K$, where *i* is the index of a particular element, and *K* is the total number of classes.

Through this process, the classifier effectively transforms raw data into a probability distribution, facilitating the interpretation of each output as a conditional probability under various scenarios. This probabilistic framework allows for a more nuanced and accurate classification, pivotal in the intricate task of human presence feature recognition.

### Ethics approval

This article does not contain any studies with human participants performed by any of the authors.

## Experiment and analysis

### Experimental setup

In response to the necessity for detailed experimental methodologies and architectural insights, this document meticulously delineates the experimental procedures, ensuring lucidity and reproducibility.

**Figure 2:**
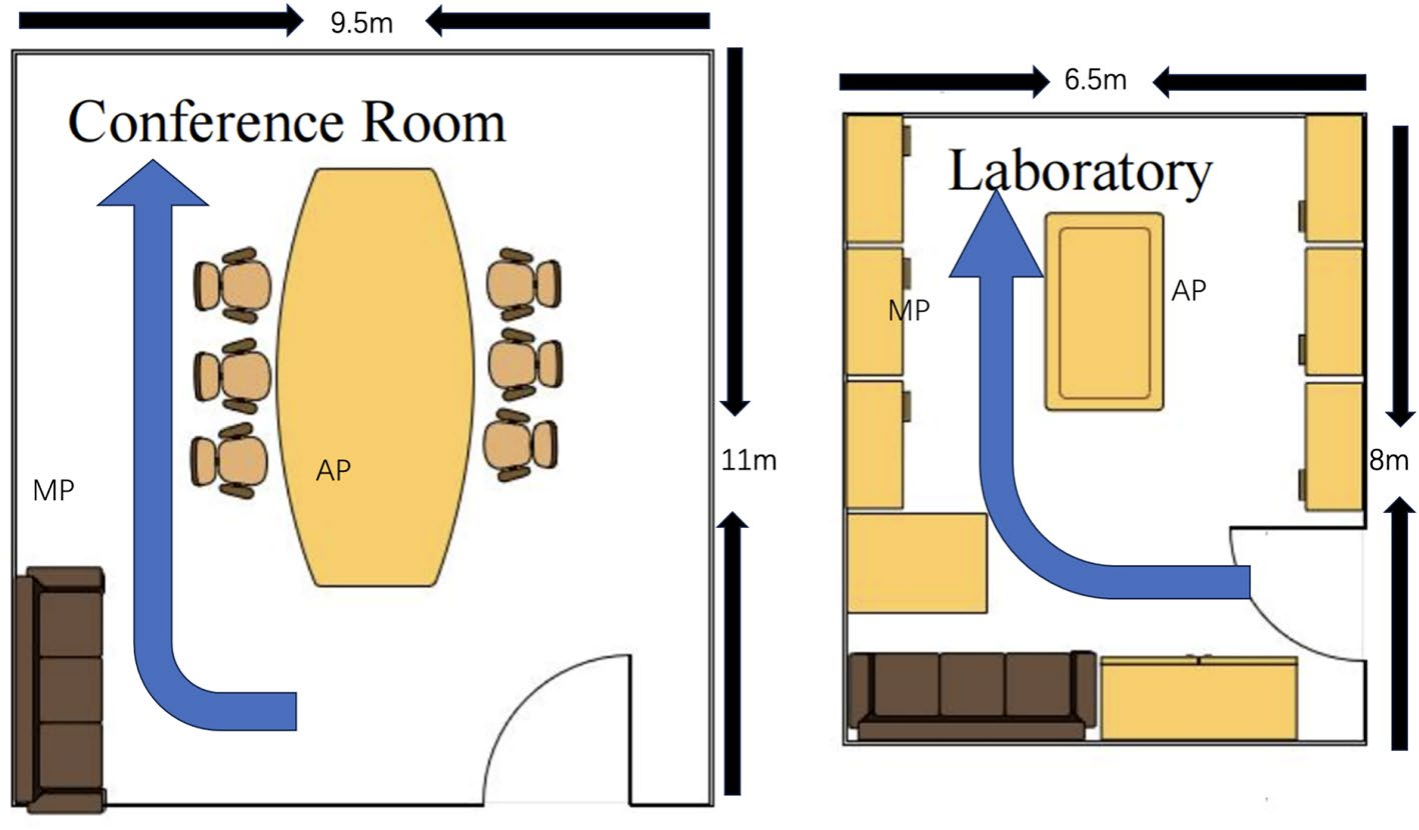
Experimental environment plan.

**Experimental setup:** The CSI dimension in our study is 1*3*30. This configuration comprises 1 transmitting antenna and 3 receiving antennas. The experimental hardware involves two Lenovo desktop computers, each powered by Intel Core i5800 CPUs. The transmitting computer is equipped with a single antenna, while the receiving computer features three antennas. The wireless transceiving system includes a Monitor Point (MP) for signal reception and an Access Point (AP) for signal transmission. The spatial arrangement of these antennas is illustrated in Fig. 2.

**Software and model construction:** The model is developed on MATLAB, a platform well-regarded for its robust capabilities in algorithm development and simulation. To ensure a consistent operational environment, both computers utilize Ubuntu 14.04 LTS. Equipped with Intel 5300 network cards, these systems are subjected to precise kernel and driver configurations prior to the installation of the CSI toolbox, as referenced in^23^. This toolbox is instrumental in processing the CSI, offering a comprehensive suite of functionalities for analyzing and interpreting wireless signal characteristics. It facilitates the extraction and manipulation of CSI data, essential for our research. Moreover, the Self-Organizing Map neural network employed in our study is configured with a default output dimension of 100, optimizing the performance for our specific application.

**Data collection environment and process:** Data was gathered in two distinct environments to evaluate the model’s versatility and efficacy under various conditions. The first environment was a laboratory measuring 6.5 m by 8 m, filled with test benches, chairs, and computers, presenting significant obstructions and strong multipath interference. The second was a spacious conference room, sized 9.5 m by 11 m, where multipath interference was minimal. In each setting, 200 datasets representing both occupied and unoccupied states were meticulously collected, with each session lasting 180 s and involving the transmission of 100 Channel State Information packets per second. The data was collected at different speeds and by different personnel, with a training and testing set ratio of 8:2.

**Dataset and validation:** Due to the lack of a standardized dataset for human presence detection, our research utilizes a proprietary dataset for model validation. This dataset covers two distinct scenarios, facilitating a thorough evaluation.

**Evaluation metrics:** To comprehensively evaluate the performance of our experiment, we utilized the True Positive Rate (TPR) and False Positive Rate (FPR) as key metrics. The True Positive Rate refers to the probability of successfully detecting the presence of a person in the test set when someone is actually present. Conversely, the False Positive Rate indicates the probability of incorrectly identifying the presence of a person when, in reality, no one is present.

### Baseline

To benchmark our model, the dataset is also utilized in replicating the methodologies outlined in the references FreeSense, Wi-alarm, and HAR.

**FreeSense**^17^: FreeSense identifies human motion by detecting the phase difference of amplitude waveforms on multiple antennas.

**Wi-alarm**^18^: Wi-alarm uses raw channel state information for human motion monitoring and uses SVM for detection.

**HAR**^21^: In this study, the author used CNN to perform edge detection on CSI data, enhancing human activity recognition based on WiFi.

### Performance evaluation

**Figure 3:**
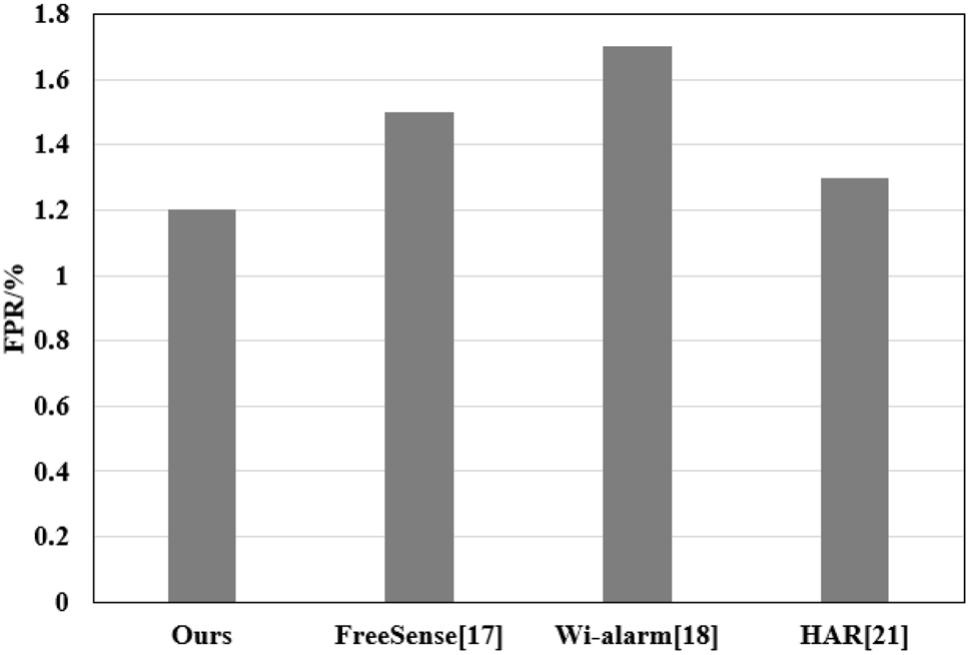
Overall performance evaluation FPR.

**Figure 4:**
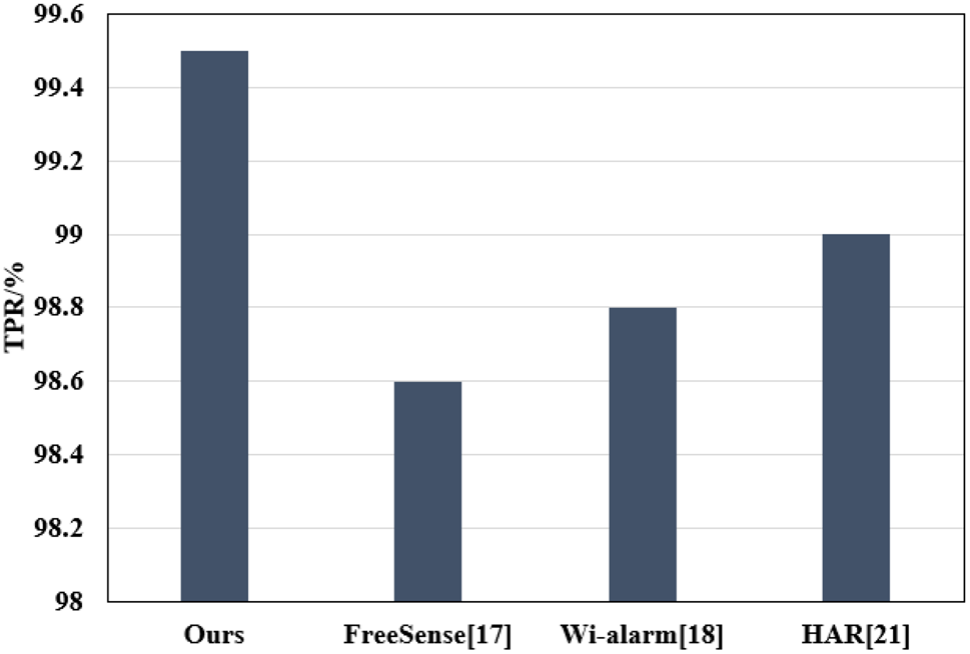
Overall performance evaluation TPR.

**Overall performance evaluation**: To effectively assess its overall performance, the method was compared with FreeSense^17^, Wi-alarm^18^, and HAR^21^. As shown in Figs. 3 and 4, the method achieved the lowest FPR value across different data types, averaging approximately 1.2$\%$, with a high TPR of 99.5$\%$. This indicates fewer false alarms triggered by the method when detecting human presence.

**Figure 5:**
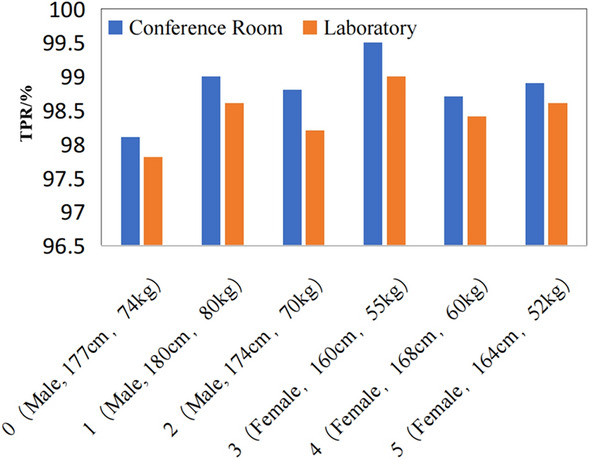
FPR under different environment and personnel posture.

**Performance analysis in different environments**: The versatility of our approach was further substantiated through a series of experiments conducted in diverse environments and postures, as depicted in Fig. 5. These figures illustrate the influence of environmental conditions on performance metrics. In both conference room and laboratory settings, the average TPR for detecting human presence was recorded at 98.8$\%$ and 98.4$\%$, respectively, while the average FPR was 1.3$\%$ and 1.5$\%$. This indicates a minor variance in the accuracy of human perception technology across different testing environments. The more spacious conference room experienced a reduced multipath effect. Conversely, the laboratory, smaller in size and cluttered with numerous objects, exhibited stronger multipath interference, leading to a decline in signal quality. Nevertheless, the overall TPR consistently exceeded 96$\%$. Notably, the SNR in the conference room was approximately 19.7 dB, compared to 17.4 dB in the laboratory environment, underscoring the method’s resilience under varying conditions.

**Performance analysis across different body types**: It allowed for the perception of individuals with varying genders, weights, and heights. The experimental results showed no substantial changes due to the posture of the subjects, thereby emphasizing the strong versatility of the method. As shown in Fig. 5.

**Figure 6:**
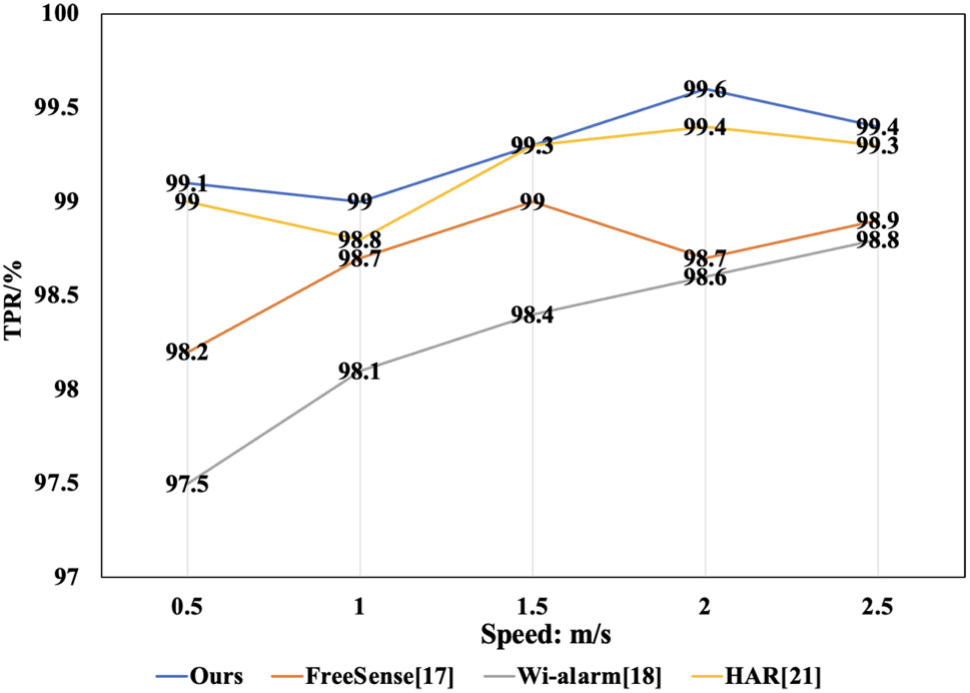
TPR at different moving speeds.

**Figure 7:**
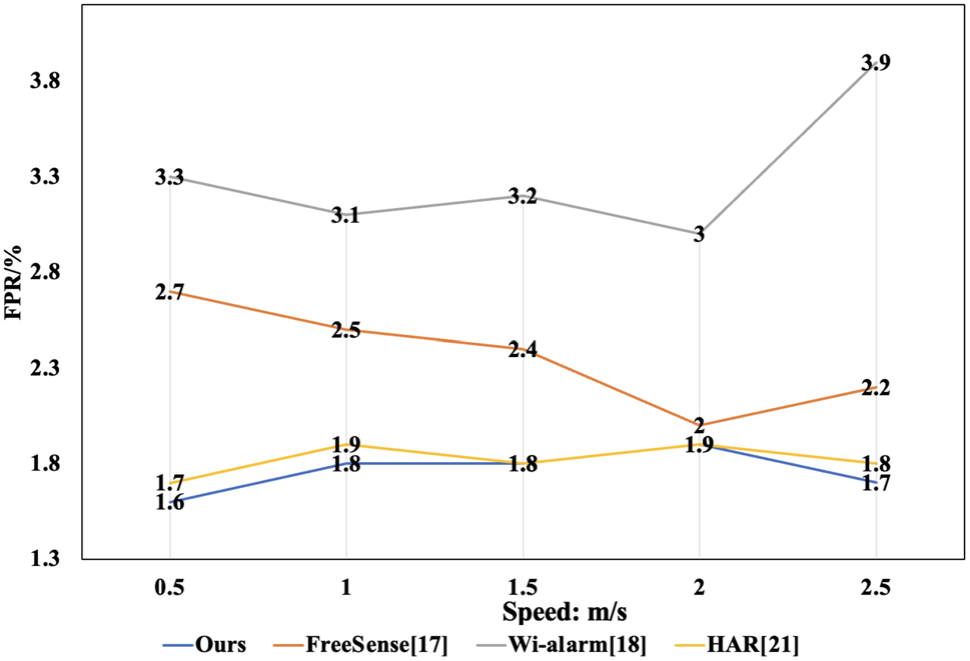
FPR at different moving speeds.

**Performance at different movement speeds**: This study also assessed human motion perception at different speeds, such as slow walking, normal walking, fast walking, and sprinting. The experimental results were compared side by side with two typical methods, as shown in Figs. 6 and 7. The findings indicated that the method maintained a stable TPR and FPR across different movement speeds. However, the FPR significantly increased while the TPR notably decreased for the other three methods when participants moved at slower speeds. This outcome can be attributed to the reduced interference characteristics of the wireless signal during the slower speed sampling period, causing less impact of the human body on the wireless signals. Nevertheless, the Self-Organizing Competitive Neural Network used in this method, with its rich feature set, maintained stable TPR and FPR, proving the robust reliability of the method.

**Performance under environmental changes**: To investigate the robustness of our model in the face of environmental changes, especially the effect of furniture rearrangement in rooms, we conducted targeted experiments. We modified the placement of chairs and tables in both lab and conference room settings to evaluate the impact on model performance. The results revealed that furniture rearrangement indeed influenced the model’s performance. In the lab setting, post-adjustment, the TPR decreased by about 1.8%, and in the conference room, it decreased by approximately 2.1%. These findings suggest that while the model demonstrates considerable robustness, it is still somewhat affected by changes in furniture layout. Importantly, these tests were conducted without retraining the model, underscoring its adaptability to environmental shifts. However, the impact of furniture layout on performance warrants attention. In future research, we aim to explore this issue more thoroughly, with the goal of developing a more stable and efficient model for human presence detection. Through optimization and adjustments, we aspire to improve its detection accuracy and robustness in diverse settings, enhancing its applicability in smart homes, healthcare monitoring, and other scenarios.

## Conclusion

Addressing the stability issues and user inconveniences of traditional human perception recognition techniques, this study presents a non-contact human presence detection technology. By preprocessing, extracting features, and classifying the CSI signals, we can discern different states such as an empty room, a room with a present individual, and a room where someone has recently been, demonstrating robust accuracy and versatility. Nevertheless, the current study only detects human presence within a room and does not recognize specific movements or simultaneous actions of multiple people, which limits its applicability. Future research will pivot towards the perception of multiple individuals’ actions, broadening the scope and functionality of this technology.

## Data Availability

The datasets used and/or analysed during the current study available from the corresponding author on reasonable request.

## Code availability

The code can be obtained from the corresponding author upon a reasonable request.
